# Dysregulated brain-gut axis in the setting of traumatic brain injury: review of mechanisms and anti-inflammatory pharmacotherapies

**DOI:** 10.1186/s12974-024-03118-3

**Published:** 2024-05-10

**Authors:** Mahmoud G. El Baassiri, Zachariah Raouf, Sarah Badin, Alejandro Escobosa, Chhinder P. Sodhi, Isam W. Nasr

**Affiliations:** Pediatric Surgery, Department of Surgery, Johns Hopkins University School of Medicine, Baltimore, MD 21287 USA

**Keywords:** TBI, Brain-gut axis, Intestinal inflammation, Microbiome, Enteroendocrine cell

## Abstract

Traumatic brain injury (TBI) is a chronic and debilitating disease, associated with a high risk of psychiatric and neurodegenerative diseases. Despite significant advancements in improving outcomes, the lack of effective treatments underscore the urgent need for innovative therapeutic strategies. The brain-gut axis has emerged as a crucial bidirectional pathway connecting the brain and the gastrointestinal (GI) system through an intricate network of neuronal, hormonal, and immunological pathways. Four main pathways are primarily implicated in this crosstalk, including the systemic immune system, autonomic and enteric nervous systems, neuroendocrine system, and microbiome. TBI induces profound changes in the gut, initiating an unrestrained vicious cycle that exacerbates brain injury through the brain-gut axis. Alterations in the gut include mucosal damage associated with the malabsorption of nutrients/electrolytes, disintegration of the intestinal barrier, increased infiltration of systemic immune cells, dysmotility, dysbiosis, enteroendocrine cell (EEC) dysfunction and disruption in the enteric nervous system (ENS) and autonomic nervous system (ANS). Collectively, these changes further contribute to brain neuroinflammation and neurodegeneration via the gut-brain axis. In this review article, we elucidate the roles of various anti-inflammatory pharmacotherapies capable of attenuating the dysregulated inflammatory response along the brain-gut axis in TBI. These agents include hormones such as serotonin, ghrelin, and progesterone, ANS regulators such as beta-blockers, lipid-lowering drugs like statins, and intestinal flora modulators such as probiotics and antibiotics. They attenuate neuroinflammation by targeting distinct inflammatory pathways in both the brain and the gut post-TBI. These therapeutic agents exhibit promising potential in mitigating inflammation along the brain-gut axis and enhancing neurocognitive outcomes for TBI patients.

## Background

TBI is a public health concern in the United States, with approximately 190 Americans dying each day from TBI-related injuries [1]. Despite its well established clinical course, the underlying mechanisms are yet to be fully elucidated [2]. TBI significantly increases the risk of morbidity and mortality, contributing to various neurodegenerative and psychiatric disorders, including Alzheimer’s, Parkinson’s, chronic traumatic encephalopathy, depression and epilepsy [3]. Recently, the gut has emerged as a crucial player in the pathogenesis of TBI, where TBI has been shown to induce detrimental changes along the GI tract. These changes ultimately worsen brain inflammation and cognitive outcomes via the gut-brain axis [4]. In this review, we will explore the mechanisms of brain-gut axis dysfunction post-TBI, with a specific focus on the role of various anti-inflammatory pharmacotherapies capable of breaking this dysregulated cycle between the brain and the gut.

## Impact of TBI on brain neuroinflammation

TBI is a form of acquired brain injury resulting from any external mechanical insult disrupting its structural integrity. TBI is primarily categorized into primary and secondary injuries. The irreversible primary injury results from blunt or penetrating trauma such as falls, motor vehicle accidents and gunshot wounds, followed by a secondary dysregulated inflammatory response that exacerbates tissue damage and neuronal injury [5]. TBI leads to a wide spectrum of consequences, ranging from immediate clinical deterioration to more severe cognitive outcomes. Clinical manifestations involve focal and diffuse brain swelling, vasospasm [6], hemodynamic perturbations (hypotension [7], hypoxia [8, 9]), metabolic derangements (hypoglycemia/hyperglycemia) [10], increased intracranial pressures (ICP) and coagulopathy disorders [11]. Moreover, TBI induces acute and chronic cognitive disorders, characterized by attention-deficits, memory problems and executive dysfunction [12]. At the cellular level, the rapid release of damage-associated molecular patterns (DAMPs) shortly after TBI initiates a dysregulated neuroinflammatory response. This involves the activation of resident brain cells (microglia, astrocytes, oligodendrocytes, neurons) and the recruitment of various immune cells into the injury site (monocytes, neutrophils, B and T-cells). DAMPs bind to “pattern recognition receptors”, such as nucleotide-binding oligomerization domain-like receptors and toll-like receptors, leading to an increase in inflammatory chemokines, cytokines and reactive oxygen species (ROS). This triggers a positive feedback loop that intensifies the inflammatory response by recruiting additional immune cells to the injury site [13, 14]. The hallmark features of TBI include neuronal injury, axonal damage, mitochondrial dysfunction, excitotoxicity, oxidative stress, and blood–brain-barrier (BBB) disruption [15, 16]. BBB breakdown occurs due to endothelial cell death, degradation of tight junction proteins, basement membrane damage, redistribution of aquaporin 4 (AQP4) channels, and swelling of astrocytic endfeet, leading to brain edema and increased ICP [17]. Excitotoxicity involves the heightened release of glutamate into the extracellular space, followed by the entry of calcium (Ca^2+^) into the cells, initiating a programmed cell death [18]. Additionally, the imbalance between increased metabolic demands and decreased mitochondrial adenosine triphosphate (ATP) production further aggravates the inflammatory response. Mitochondrial dysfunction results from a combination of increased ROS, Ca^2+^ overload and excitotoxicity, in addition to alterations in the expression of caspases, B-cell lymphoma 2 (Bcl-2) family proteins and apoptosis inducing factors [19]. TBI has also been shown to increase levels of polyunsaturated fatty acids within the brain, cerebrospinal fluid and serum in both preclinical and clinical models [20, 21]. These fatty acids activate the arachidonic acid metabolic pathways, leading to the release of prostaglandins and leukotrienes, further contributing to brain injury [22].

Astrocytes and microglia are key cells in the CNS that can initiate the inflammatory response post-TBI [13]. These cells have the ability to acquire proinflammatory or anti-inflammatory phenotypes and can secrete various chemokines, cytokines and growth factors [23, 24]. These changes influence the local tissue microenvironment and modulate secondary cellular damage or tissue repair [25]. Astrocytes undergo a process called reactive astrogliosis, which involves molecular, structural and functional changes [26]. They regulate the neuroinflammatory responses, scar formation, blood–brain barrier permeability and synapse remodeling [27]. At the same time, activated microglia can migrate toward the lesion site to phagocytose debris and modulate the inflammatory profile by secreting various cytokines and chemokines [28]. Microglia can have distinct roles in neurodegeneration and tissue repair depending on their activation state (pro/anti-inflammatory) [29]. Proinflammatory microglia favor the production of cytokines that exacerbate neural injury, while the anti-inflammatory microglia acquire a phagocytic role and promote repair by releasing neurotrophic factors [30, 31]. Recently, their role has expanded to show that changes in the gut microbiome after TBI can also alter microglial phenotypes in the brain and ultimately influence TBI outcomes [32]. The chronic activation of both astrocytes and microglia leads to increased peripheral immune cell infiltration through the permeable BBB, further exacerbating cognitive outcomes and increasing risk of morbidity and mortality [33].

## Brain-gut axis disruption following TBI

**Fig. 1:**
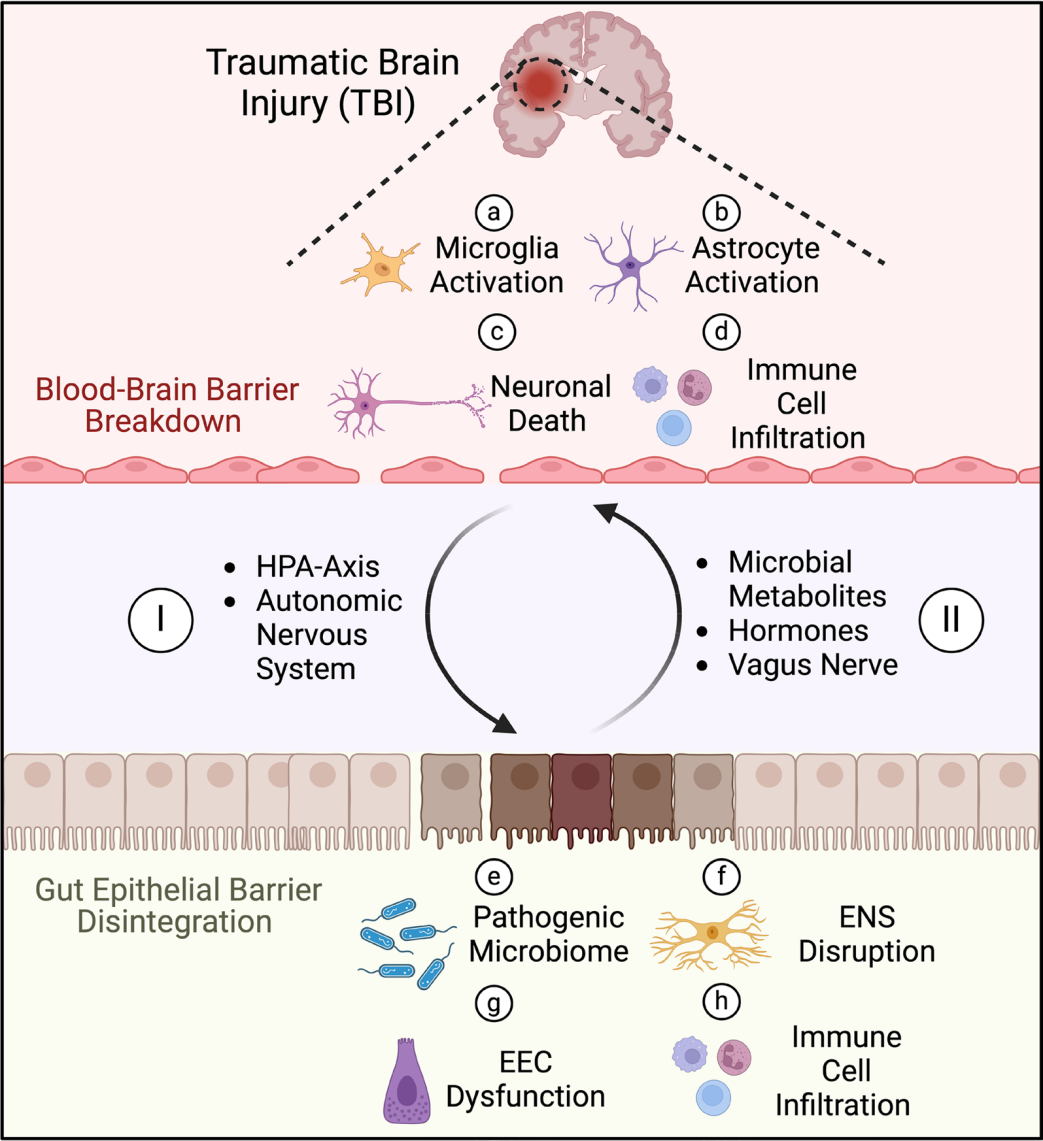
Bidirectional cross-talk between the brain and gut in TBI. TBI induces tissue and cellular disruption, leading to the release of various inflammatory cytokines, chemokines, complement factors and damage-associated patterns (DAMPs), which promote diverse cellular responses: **a** Microglia, the resident immune cells of the brain, become activated and migrate towards the injury site to phagocytose debris and release proinflammatory cytokines. **b** Astrocytes contribute to the inflammatory response and undergo reactive gliosis, leading to the formation of a protective glial scar to limit injury spread. **c** Increased production of reactive oxygen species (ROS), excessive release of excitatory glutamate, and reduced blood flow to the brain promotes neuronal apoptosis and neurodegeneration. **d** Blood–brain barrier breakdown leads to the infiltration of various immune cells, such as neutrophils, monocytes and T-cells, into the brain parenchyma to aid in debris clearance. However, excessive infiltration can exacerbate tissue damage and worsen neurologic outcomes. **I** TBI affects the gut through several pathways, including the activation of the hypothalamic–pituitary–adrenal (HPA) axis, which releases cortisol, and sympathetic arm of the autonomic nervous system (ANS), which releases catecholamines, leading to gut intestinal barrier disintegration. **e** This allows the translocation of pathogenic bacteria from the gut lumen into the intestinal parenchyma, exacerbating microbial dysbiosis. **f** The enteric nervous system (ENS) becomes dysfunctional, with reactive gliosis in enteric glial cells, leading to dysmotility. **g** These changes also result in decreased expression of enteroendocrine cells (EECs), reducing their secretion of anti-inflammatory hormones such as serotonin. **h** Inflammatory immune cells, including T-cells and monocytes, infiltrate the intestinal epithelium and further increase gut inflammation. The gut sends signal back to the brain through various pathways: (II) Decreased release of microbial metabolites such as short-chain fatty acids (SCFAs) and bile acids, as well as reduced anti-inflammatory hormone secretion from EECs, worsen this detrimental cycle. Furthermore, the impairment of afferent and efferent vagus nerve pathways disrupts brain-gut homeostasis and exacerbate the neuroinflammatory response in the injured brain. These overlapping and interrelated pathways offer potential therapeutic targets for mitigating TBI-induced neuroinflammation and improving neurologic outcomes. Created with www.Biorender

TBI is a systemic disease that impacts various peripheral organs, including the lungs, GI tract, liver and kidneys [34–37]. TBI survivors have an increased risk of death from septicemia [38], pneumonia [39] and digestive diseases [40] compared to their healthy counterparts. Furthermore, TBI induces gastroparesis and intestinal dysmotility, resulting in feeding intolerance, where 50% of patients with severe TBI experience feeding intolerance within the first week after injury [41]. A single-center observational study conducted by McConnochie et al. demonstrated that around 52% of TBI patients often experience delayed bowel defecation, which was associated with longer intensive care unit (ICU) stays and higher gastric residual volumes [42]. A retrospective study also revealed that patients with TBI are 2.5 times more likely to die from digestive disease-related conditions and 12 times more likely to die from septicemia compared to the general population [43]. Another retrospective study demonstrated rates of fecal incontinence up to 70% in patients with acquired brain injury admitted to rehabilitation centers [44]. Fecal incontinence also significantly correlated with the presence of frontal lobe lesions and hemodynamic instability [45]. Furthermore, abdominal pain, distention and constipation were also common symptoms in patients even two years after their initial brain injury [46]. Therefore, current guidelines recommend initiating enteral nutrition within 24–48 h to attenuate TBI-induced intestinal dysfunction and improve patient prognosis [47]. Various distinct and overlapping pathways are also involved in TBI-induced intestinal dysfunction, including systemic immune dysregulation, ANS dysfunction, intestinal flora dysbiosis and neuroendocrine system disruption (Fig. 1).

### Systemic immune dysregulation

TBI detrimentally affects the GI tract through systemic pathways by modulating the peripheral immune system [48]. TBI triggers the activation of the hypothalamic–pituitary–adrenal (HPA) axis and sympathetic nervous system, resulting in elevated levels of glucocorticoids/cortisol and catecholamines, respectively [49]. This surge in cortisol can ultimately lead to a leaky gut by increasing the intestinal barrier permeability [50, 51]. Subsequently, the translocation of luminal pathogenic bacteria across the disintegrated barrier can induce a systemic inflammatory response syndrome (SIRS) and further aggravate systemic inflammation by releasing numerous cytokines and chemokines into the systemic circulation [52]. TBI leads to an acute increase in proinflammatory mediators such as TNF-α, IL-1β and IL-6, which have been associated with worse cognitive outcomes [53]. Elevated levels of IL-1β were linked to impaired working memory in the acute phase, while increased levels of chemokine ligand 2 (CCL2) correlated with greater severity of post-concussive symptoms [54]. Further studies demonstrate that an increase in cytokine score load shortly after injury correlates with unfavorable Glasgow Coma Scale (GCS) scores at six and twelve months post-TBI [55]. Moreover, elevated blood cortisol levels can elevate the risk of infections by inducing peripheral immunosuppression [56]. TBI results in increased levels of circulating neutrophils and a decrease in circulating monocytes, T-cell lymphocytes and natural killer (NK) cells, accompanied by defective phagocytosis [57, 58]. This is characterized by impairments in respiratory burst and phagocytosis in neutrophils and monocytes, as well as decrease in percentage of perforin-positive NK cells [59, 60]. Additionally, TBI induces thymic involution, leading to chronic T-cell lymphopenia [59]. These alterations result in a shift in systemic immunity towards an anti-inflammatory state, which could explain the increased risk of nosocomial infections in hospitalized patients following TBI [61]. While these changes may be necessary to combat TBI effects acutely, the chronic stress response leads to a prolonged hyperinflammatory state that can trigger multi-organ dysfunction and, ultimately, lead to death [62, 63]. Interestingly, TBI has also been shown to increase age-related microglial phenotypes, as depicted by lipid accumulation in microglia for up to one year after injury [64]. All these acute, subacute and chronic changes in the neuroimmune responses exacerbate inflammation and accelerate immune aging and neurodegeneration [65].

To assess the impact of the systemic immune system on gut inflammation following TBI, our group have demonstrated that knocking out peripheral inflammatory C–C chemokine receptor type 2 (Ccr2)-dependent monocytes reduce levels of intestinal tumor necrosis factor alpha (TNF-α), interleukin-1β (IL-1β) and lipocalin-2. Additionally, TBI induces a significant increase in the expression of intestinal toll-like receptor 4 (TLR4) shortly after injury, which could explain aggravated intestinal inflammation. Notably, Ccr2^ko^ mice showed decreased levels of intestinal TLR4 on days 1 and 3 post-injury [66]. These findings suggest a critical role for the systemic innate immune system in modulating the bidirectional communication between the brain and the gut post-TBI. In addition to the innate immune system, adaptive immunity, especially T-cells, has been shown to modulate the neuroinflammatory response along the brain-gut axis [67]. T-cells infiltrate the brain as early as 5 days post-TBI [68], and granzyme^+^ CD8^+^ T-cells have also been detected 8 months after TBI [69]. Daglas et al. demonstrated that pharmacologic and genetic depletion of CD8^+^ T-cells improves neurologic outcomes and produces a neuroprotective Th2/Th17 immunologic shift [69]. Recent studies have also shown that the gut microbiota modulate the trafficking of effector T-cells from the gut to the leptomeninges, impacting injury outcomes in both stroke and TBI models [70]. T-cell trafficking is thought to be modulated by changes in the microbiome following CNS injury, which ultimately regulates microglial functions in the brain [71]. Further research is warranted to understand the role of intestinal innate and adaptive immunity, particularly macrophages/monocytes and T-cells, in modulating neurocognitive outcomes post-TBI.

### Autonomic and enteric nervous system dysfunction

In addition to the HPA-axis, the CNS exerts its effects on the gut through branches of the ANS. All divisions of the ANS innervate the GI tract, including the parasympathetic nervous system, sympathetic nervous system as well as the intrinsic ENS [72]. The bidirectional brain-gut axis serves as a conduit through which ANS exerts its influence on the ENS. TBI is associated with sympathetic hyperactivity, resulting in a surge of circulating catecholamine levels that significantly impact various peripheral organs, particularly the GI tract [73]. Increased catecholamine levels persist for weeks after injury and play a critical role in inducing systemic immunosuppression, exacerbating clinical outcomes, and increasing morbidity and mortality rates [74]. TBI induces a sympathetic storm of systemic epinephrine, which redirects blood away from the GI tract, resulting in gut dysmotility and gastroparesis [75]. Elevated catecholamine levels in the gut also disrupt ENS homeostasis, which is primarily cholinergic in nature [76]. A study by Ma et al. demonstrated ENS dysregulation four weeks post-TBI, depicted by an increased activation of enteric glial cells (EGCs) in the colon. This process, known as reactive gliosis, may further contribute to intestinal dysmotility [77]. As for the parasympathetic nervous system, the vagus nerve is the major regulator of bidirectional neuroimmune interactions between the brain and the gut [78]. The vagus nerve sends signals from the brain to the gut through postganglionic efferent neurons that regulate the secretomotor function of the gut by secreting Acetylcholine (Ach), thereby promoting gut motility [79]. Furthermore, Ach also binds to the α7 subtype of the nicotinic acetylcholine receptor (α7nAChR) located on intestinal macrophages and decreases the production of inflammatory cytokines [80]. Recent findings have also highlighted that extracellular choline acetyltransferase (ChAT), the rate limiting enzyme in Ach biosynthesis, reduces systemic inflammation and inhibits the release of proinflammatory cytokines. This effect was observed following vagus nerve stimulation or administration of a bioactive recombinant form of ChAT, as evidenced by reduced levels of serum proinflammatory markers TNF-α and IL-6 in a DSS colitis model in mice, two weeks post-injury [81]. In turn, the vagus nerve carries information from the gut to the brain through its afferent neurons which regulate brain neuroinflammatory outcomes. Visceral afferents respond to various mechanical and chemical stimuli [82], including changes in microbiome diversity [83], neuropeptides and hormones secreted by enteroendocrine cells (EECs) [84], as well as sensitization to inflammatory mediators. The importance of vagus nerve function is highlighted in vagus nerve stimulation (VNS) preclinical trials, which demonstrated improvements in cognitive outcomes following TBI when compared to placebo [85]. Therefore, dysautonomia plays a crucial role in mediating TBI outcomes post injury, and therapies should be targeted at attenuating the sympathetic storm with the potential use of beta-blockers. Additionally, there should be a greater focus on stimulating the parasympathetic vagus arm and studying its impact on immune cellular responses in the brain and gut, in addition to functional outcomes including intestinal permeability, gut motility and neurobehavioral outcomes.

### Intestinal flora dysbiosis

The impact of the gut microbiota on behavioral outcomes and cognition is a rapidly expanding field of research, suggesting that alterations in the gut play a crucial role in the pathophysiology of TBI [86]. The impact of TBI on microbiome diversity and richness has been extensively studied in the preclinical [87, 88] and clinical settings [89, 90]. TBI leads to an increase in pathogenic bacteria and a decrease in protective populations [91]. These changes have been shown to manifest as early as 2 h post-TBI [92] and can persist for years [89] after the initial injury. Various factors could contribute to microbial dysbiosis, including changes in intestinal motility and alterations in paneth cells expression. Reduced gut peristalsis shifts microbial composition into a pathogenic state, and this in turn can worsen gut motility, triggering a detrimental cycle between dysmotility and dysbiosis [93]. Additionally, Paneth cells are another type of epithelial cells found the in the intestinal crypts that secrete various antimicrobial peptides, including lysozyme and α-defensins [94]. They have been also shown to regulate the composition of bacterial microbiome in various intestinal diseases such as inflammatory bowel disease (IBD) and irritable bowel syndrome (IBS) [95]. In the setting of TBI, Yang et al. demonstrated a significant correlation between decreased expression of lysozyme antimicrobial peptides and increased translocation of pathogenic microbiome across the disintegrated epithelial barrier [96]. These changes suggest a critical role for paneth cells in modulating the microbiota-gut-brain axis following TBI.

The influence of gut microbiota on the brain is mediated mainly through vascular, neural and immune pathways. TBI results in mucosal damage and increased epithelial barrier permeability which facilitates the translocation of luminal pathogenic bacteria into the intestinal parenchyma [96]. The intestinal microbiota also transforms dietary components into various metabolites including short-chain fatty acids (SCFAs), tryptophan metabolites, and bile acid metabolites [97]. These microbial metabolites can either influence the brain directly by binding to receptors on vagal afferents, or indirectly by entering the systemic circulation and modulating cell–cell interactions between gut microbes and cells in the central nervous system (CNS), primarily astrocytes, microglia and neurons [98, 99]. In a study Xiong et al., it was demonstrated that administrating SCFAs such as butyrate and acetate for six months following TBI reduced microglial activation and promoted an anti-inflammatory microglial phenotype, while also attenuating T-cell activation and cytotoxic-related pathways [100]. Similarly, the administration of antibiotics, specifically amoxicillin-clavulanic acid, was linked to decreased infiltration of T-cells into the brain two days after TBI [70]. The interactions of various microbiome metabolites and brain immune cells such as microglia and T-cells, may ultimately impact cognition and neurobehavioral outcomes. Therefore, therapies targeted towards promoting a healthy microbiome are essential in ameliorating the hyperinflammatory response along the brain-gut axis and improving cognitive outcomes following TBI.

### Neuroendocrine system disruption

**Fig. 2:**
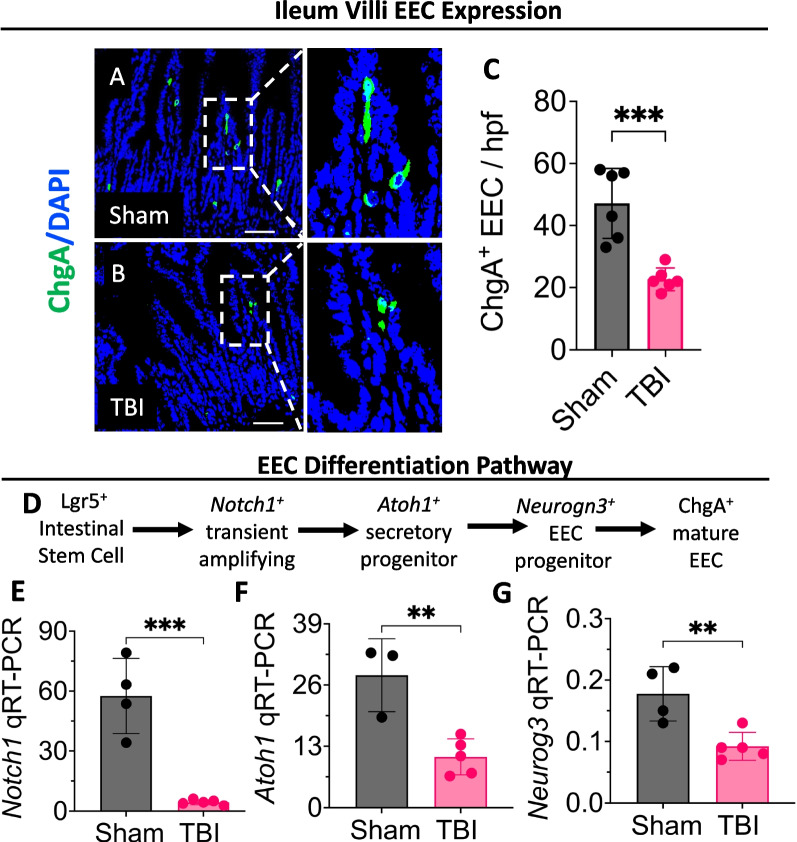
TBI induces enteroendocrine cell loss and decreases EEC differentiation. male C57BL/6 mice were used at the age of 4–6 weeks to induce moderate-severe TBI as previously described [66]. Mice were sacrificed three days later, and the ileum was harvested to investigate the expression of EECs. Intestinal tissues were fixed overnight with 4% paraformaldehyde and processed for paraffin embedding. Subsequently, 5-μm tissue sections were cut from paraffin blocks using a CUT 6062 microtome (SLEE Medical GmbH, D-55129 Mainz, Germany) and stained for DAPI (blue) and chromogranin A (ChgA) (green). ChgA^+^ cells were counted using ImageJ2 software. Scale bars, 50-μm. **A**–**C** Representative confocal images showing decreased intestinal ChgA expression in TBI mice when compared to sham on post-injury day (PID) 3. **D** An illustration of EEC differentiation pathway in the intestine. TBI reduces the expression of key transcription factors implicated in the differentiation of Lgr5^+^ intestinal stem cells into ChgA^+^ mature EECs, including **E** *Notch1*, **F** *Atoh1*, and **G** *Nuerog3*, as measured by quantitative reverse transcription polymerase chain reaction (qRT-PCR). The mRNA levels are expressed as relative to housekeeping gene *Rplp0* expression. Statistical significance was determined by student’s t-test using GraphPad Prism 10 software. Each dot on the graph represents a different mouse. Error bars indicate the mean ± SEM. *p < 0.05, **p < 0.01, ***p < 0.001

Previous research focused on investigating brain-gut bidirectional communication following TBI, emphasizing the role of the HPA-axis, ANS, cellular immune responses and the microbiome axis. However, a thorough examination of the neuroendocrine axis is warranted, given the gut’s capacity to release a diverse array of hormones that can ultimately influence cognitive outcomes [101]. EECs are specialized cells lining the intestinal epithelium which form the largest endocrine system in the body. They play a pivotal role orchestrating the crosstalk between the brain and the gut by releasing various hormones and peptides including 90% of the body’s serotonin, ghrelin and glucagon-like peptide 1 (GLP-1), neuropeptide Y and cholecystokinin [101, 102]. EECs can also influence the brain directly through excitatory synaptic connections with the vagus nerve [103] and/or indirectly by releasing various hormones into the systemic circulation [104], thereby forming an interface between the brain and the gut. Our group was the first to show substantial reductions in the expression of EECs three days following TBI as demonstrated by a significant downregulation in the expression of chromogranin A (ChgA), which is the main marker of EECs (Fig. 2A–C). We have also shown reduction in the transcription factors implicated in the differentiation of Leucine-rich repeat-containing G-protein coupled receptor 5 (Lgr5^+^) intestinal stem cells into mature ChgA^+^ cells, including notch receptor 1 (*Notch1)*, atonal bHLH transcription factor 1 (*Atoh1)*, and neurogenin 3 (*Neurog3)* (Fig. 2D, E). The involvement of EECs in brain-gut communication has been also studied in various neurologic and psychiatric disorders such as Parkinson’s disease and schizophrenia [105, 106]. In Parkinson’s, EECs have been shown to express the α-synuclein misfolded proteins which directly connect to α-synuclein–containing nerves, forming a neural circuit between the gut and the nervous system [106]. More research is expected down the line to study the impact of EEC changes during acute and chronic timepoints post-TBI, alongside the various hormones they produce especially serotonin, ghrelin and GLP-1 which are primarily characterized as anti-inflammatory agents.

**Fig. 3:**
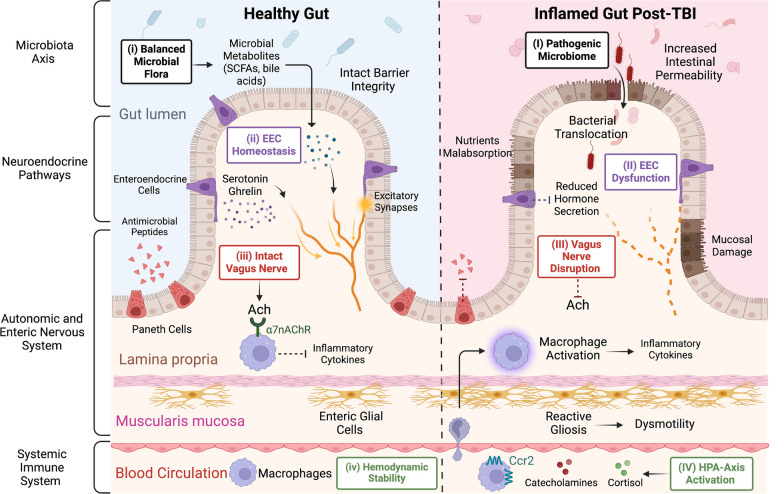
TBI induces gut dysfunction via the brain-gut axis. This illustration depicts the intricate pathways through which TBI disrupts gut function via the brain-gut axis. Four main pathways contribute to the cross-talk between the brain and gut, including the systemic immune system, autonomic and enteric nervous systems, neuroendocrine system, and microbiota axis. In a healthy gut (left), (i) a balanced microbial flora transforms dietary components into various metabolites, including short-chain fatty acids (SCFAs), tryptophan metabolites, and bile acids. SCFAs, particularly acetate, propionate and butyrate exert various beneficial effects on brain function by acting as energy substrates for neurons and microglia. They have the ability to directly influence the brain by entering the systemic circulation and crossing the blood–brain-barrier or indirectly by binding to receptors on vagus nerve endings; (ii) Additionally, enteroendocrine cells (EECs), the largest endocrine system in the body, regulate digestive processes by secreting various hormones, such as like serotonin, ghrelin and glucagon-like peptide 1 (GLP-1), in response to luminal stimuli. These cells communicate bidirectionally with the CNS by sending hormonal signals to the brain via the blood stream or and neural signals through vagal afferent pathways; (iii) An intact vagus nerve also releases acetylcholine (Ach), which binds to the α7-subtype of the nicotinic acetylcholine receptor (α7nAChR) located on intestinal macrophages and decreases the production of inflammatory cytokines. Ach also binds to muscarinic receptors located on smooth muscle cells in the GI tract facilitating peristalsis and Ach also stimulate excitatory motor neurons in the ENS which further enhances gut motility; (iv) In normal homeostasis, immune cell activation is balanced, cortisol secretion follows diurnal rhythms and catecholamine levels remain within physiologic ranges, collectively maintaining a stable circulation and tissue perfusion. However, TBI-induced intestinal dysfunction triggers a cascade of changes (right). (I) The microbiome shifts towards a pathogenic state, with bacteria translocating into the intestinal parenchyma through the compromised intestinal barrier; (II) EECs become dysfunctional, reducing their expression, differentiation and secretion of anti-inflammatory hormones; (III) Concurrently, vagus nerve dysfunction occurs, resulting in decreased Ach, which can polarize macrophages into a proinflammatory state and heighten inflammation; and (IV) The activation of hypothalamic–pituitary–adrenal (HPA) axis during TBI increased circulating catecholamines and cortisol, leading to a leaky gut and mucosal damage. Furthermore, systemic monocytes and T-cells infiltrate the gut and exacerbate inflammation by upregulating proinflammatory cytokines. In addition, reactive gliosis in enteric glial cells (EGCs) results in intestinal dysmotility. Finally, Paneth cells reduce their secretion of antimicrobial peptides, further exacerbating microbiome dysbiosis. These intricate changes collectively aggravate brain inflammation and neurodegeneration through the gut-brain axis. Created with www.Biorender

In summary, the bidirectional communication between the brain and gut occurs through systemic immune pathways, neural networks, endocrine hormones, and the microbiota axis, thereby inducing detrimental changes along the GI tract post-TBI. These changes further exacerbate brain injury, as illustrated in Fig. 3. Strong evidence supports the role of diverse therapeutic agents in breaking this deleterious cycle and reducing inflammation through distinct mechanisms in the brain and the gut. In this comprehensive review, we highlight the anti-inflammatory role of various therapeutic modalities, including hormones such as (1) serotonin; (2) ghrelin; (3) progesterone; ANS modulators such as (4) beta-blockers; lipid-lowering agents such as (5) statins; and intestinal flora modulators such as (6) probiotics/antibiotics. We describe how these therapeutic interventions attenuate the hyperinflammatory response along the brain-gut axis following TBI.

## Therapies attenuating brain-gut axis disruption following TBI

### Serotonin

In the brain, serotonin is produced by neurons originating in the raphe nucleus located in the brainstem. Serotonin works as a neurotransmitter known for its role in regulating mood; however, it has also been shown to be involved in other processes such as neurogenesis and plasticity [107–109], cognition [110, 111], memory [112], inflammation [113], and gut motility [114, 115]. Serotonin and kynurenine, which are upregulated in inflammatory states, are both synthesized from tryptophan and compete for its availability. Most of the tryptophan in the body is used for the synthesis of kynurenine, and only a minority is used for serotonin. Tryptophan hydroxylase (TPH) is the rate-limiting enzyme of 5-HT (serotonin) synthesis and is present both in the CNS and the peripheral organs. Serotonin cannot cross the BBB, and as such, it is synthesized via TPH1 in the EEC of the gut, which are responsible for the synthesis of 90% of the serotonin in the body and via TPH2 in the CNS [107, 112, 113, 115]. There are a total of 14 known serotonin receptors that are divided into 7 classes, 5-HT_1_ to 5-HT_7_. The receptor that 5-HT activates, its location in the body, and the concentration it activates it with, determine the effect it exerts [107, 112, 113, 115].

Altered serotonin levels have been observed in TBI patients, which could be explained by alterations in tryptophan metabolism [109]. Zhang et al. showed that a rabbit subjected to controlled cortical impact had increased inflammation and upregulation of the enzyme responsible for kynurenine synthesis, shifting tryptophan away from serotonin synthesis. Although serotonin levels were not significantly decreased, there was a significant decrease in the serotonin/tryptophan ratio, with a decrease in the mean and total length of serotonin fibers compared to sham [109]. These alterations in 5-HT levels could contribute to brain dysfunction, as cognitive impairment was demonstrated to be mediated by 5-HT in the hippocampus in a tryptophan depletion clinical trial [112]. Considering such evidence, the role of serotonin in treating or altering the outcomes of TBI is under study [66, 116, 117]. Data about the efficacy of selective serotonin reuptake inhibitors (SSRIs) in treating TBI patients comes from preclinical models. Weaver et al. showed that mice subjected to severe controlled cortical impact, followed directly by a single injection of intraperitoneal (IP) fluoxetine (5 mg/kg), had decreased colonic permeability and improved motor coordination starting day 4 post-TBI compared to those treated with placebo [117]. Craine et al. studied the role of chronic IP milnacipran treatment (30 mg/kg/day), a serotonin-norepinephrine reuptake inhibitor (SNRI), in improving cognition in male rats subjected to frontal lobe injury. After TBI, rats treated with milnacipran performed significantly better in attentional set-shifting tasks compared to those treated with placebo, and their performance was comparable to that seen in sham rats [111]. In humans, a systematic review conducted by Yue et al. demonstrated that the administration of SSRIs was associated with improvements in depressive symptoms following TBI [118, 119]. In addition to TBI, many CNS disorders have been associated with altered levels of 5-HT. For example, patients with Alzheimer’s disease (AD) were found to have decreased total levels of 5-HT and 5-HT receptors [120–122], and a decrease in 5-HT along with an increase in kynurenine was associated with increased cognitive dysfunction and inflammation [123]. Similarly, in Parkinson’s patients, 5-HT depletion was associated with worse cognitive functioning [124–126]. In healthy individuals, acute serotonin depletion achieved through tryptophan depletion has been associated with impaired verbal memory and emotional processing [127, 128]. This evidence is further supported by the impact of SSRIs in alleviating some of these conditions. Preclinical studies showed that treatment with SSRIs decreased amyloid-beta plaques in female mice with AD, which was associated with improved cognitive function, learning and memory [129]. There is also evidence that longer treatment duration with SSRIs is protective against dementia [130, 131]. The mechanism of how serotonin can improve these conditions is not fully understood, as preclinical models show that agonism of different 5-HT receptors can result in either a positive or negative impact on memory and cognition [132, 133].

Since the gut is the main source of 5-HT in the body, it is not surprising the serotonin levels are altered in TBI patients. Mercado et al. show that male mouse models subjected to moderate TBI have a significant decrease in TPH1 in the duodenum and colon by 0.9-fold and 0.5-fold, respectively. Additionally, there was a reduction in serotonin expression in the colon on immunofluorescent staining accompanied by a significant increase in colonic serotonin reuptake transporters (Sert) compared to sham. This was reflected as an overall significant decrease in 5-HT level in the peripheral circulation [116]. Furthermore, our group was the first to demonstrate a significant downregulation in the expression of EECs along the intestinal epithelium, particularly the ileum, following severe unilateral TBI in male mice. This results in decreased levels of serotonin synthesis genes, especially dopa decarboxylase, another essential enzyme responsible for serotonin synthesis [66]. The long-term gut dysbiosis that occurs because of inflammation and impaired motility in TBI is another factor that explains the changes in 5-HT levels [4, 134]. Studies involving alterations in gut microbiota whether in induced or in disease states such as IBS have been correlated with changes in 5-HT levels and in behavior [135–137]. Similarly, people who suffer from schizophrenia [138] or depression [139] harbor pathogenic microbiota and reduced 5-HT levels [140, 141] than controls highlighting the bidirectional interaction.

In the GI system, serotonin has been shown to play a role in regulating motility through 5-HT_3_ and 5-HT4, and peristalsis through its action on 5-HT_2B_ on the interstitial cells of Cajal (ICC), which are the cells responsible for initiating rhythmic contraction [112–115]. In addition to TBI and other neurological diseases, alterations in 5-HT levels have been extensively described in intestinal diseases such as IBDs [142, 143], celiac disease [144, 145], colitis [146], and diverticulitis [147]. Serotonin has been shown to improve esophageal motility in cases of dysfunction [148], increase stool frequency and improve its consistency in patients with inflammatory bowel syndrome associated with constipation [149]. Interestingly, chronic treatment with SSRIs has been associated with decreased gut motility and delayed transit both in mouse and human studies [150, 151], highlighting the complexity of serotonin signaling. 5-HT has been also shown to regulate inflammation in the gut by exerting both pro-inflammatory and anti-inflammatory effects through various receptors found on immune cells [152]. Given that intestinal epithelial cells express the serotonin reuptake transporter (Sert) on their apical membranes [153] and considering that TBI increases the expression of these receptors in the intestinal epithelium of colonic villi [116], administering SSRIs such as fluoxetine presents a promising avenue to explore the impact of intestinal serotonin on gut-brain axis interactions and neurobehavioral outcomes following TBI.

### Ghrelin

First characterized in 1999, ghrelin is an orexigenic peptide hormone mainly produced by P/D1 cells in the stomach with small amounts also released by the small intestine, pancreas and brain. Ghrelin is well known for its effects on pituitary regulation, hunger, and satiety [154–156]. More recently, its anti-inflammatory properties have become better understood [157, 158]. Investigation into the role of exogenous ghrelin in models of brain injury has uncovered several mechanisms through which ghrelin can simultaneously ameliorate GI dysfunction and exert various neuroprotective effects. The neuroprotective effects of ghrelin have been linked to BBB preservation, reduction in oxidative damage, as well as other neuroprotective mechanisms. In a weight drop (WD) model of TBI, IP administration of ghrelin (20 μg total) in male mice was found to reduce neuronal degeneration and decrease vascular permeability and perivascular expression of AQP-4 [159]. Increased AQP-4 has been shown to be linked to cellular edema following TBI [160] which contributes to TBI-induced neuronal damage. In this study, S100B -a neurobiochemical marker of brain damage- was also found to be reduced in the serum of mice that receive ghrelin alongside TBI [159, 161], adding evidence that ghrelin prevents neuronal damage and apoptosis following trauma through preservation of the BBB. The protective role of ghrelin in TBI was linked to fibroblast growth factor (FGF) where a study by Shao et al. found that both FGF-binding protein (FGF-BP) and basic FGF (bFGF) were downregulated in the ipsilateral hemisphere following treatment with IV ghrelin (20 μg/kg) in a TBI rat model. The group proposed that ghrelin attenuates brain injury by competitively inhibiting bFGF/FGF-BP-induced neovascularization. However, it remains to be investigated whether this discovered relationship between ghrelin and both bFGF and FGF-BP is causative or competitive [162]. An alternative mechanism has been identified in relation to ghrelin's protective role against brain injury during sepsis. Sun et al. found that PI3K/Akt signaling activation mediates ghrelin’s ability to attenuate brain edema, neuronal apoptosis and enhanced BBB integrity [163]. Phosphoinositide 3-kinases (PI3K) activation results in the production of phosphatidylinositol 3,4,5 trisphosphate (PIP3) and phosphatidylinositol 3,4 bisphosphate (PIP2), leading to the activation of Protein kinase B (Pkb/Akt). Akt promotes cell survival by phosphorylating both glycogen synthase kinase 3beta (GSK-3beta) and the Bcl-2 family [164, 165]. These two molecules are tied closely to neuronal survival, highlighting a potential mechanism through which ghrelin exerts its neuroprotective effects during sepsis. Lastly, ghrelin has been shown to be exhibit neuroprotective properties in cerebral models of ischemia–reperfusion injury (IRI) [166]. When compared to IRI controls, treatment with un-acylated ghrelin in rat and mice models of cerebral IRI resulted in decreased injury, apoptosis, inflammation and BBB disruption through the reduction of oxidative damage [167, 168]. Ghrelin has also been found to reduce neutrophil infiltration following spinal cord injury (SCI), resulting in less lipid peroxidation and DNA damage following the insult [169, 170]. However, despite these potential protective roles, investigation into ghrelin has not yielded universally positive findings. For example, Ersahin et al. found that IP ghrelin (10 μg/kg/day) did not reduce neurological deterioration following SCI in rats [169]. This divergence in available data emphasizes the need for further investigation into the potential role of ghrelin as a protective agent that acts on and through the brain-gut axis.

Ghrelin’s ability to reduce GI dysfunction following CNS injury has been attributed to several mechanisms, including a link with the vagus nerve, preservation of the intestinal barrier, and activation of the mammalian target of rapamycin (mTOR) pathway following ischemic injury. Evidence supports the role of ghrelin in mediating protective responses of the gut-brain axis following TBI. Bansal et al. showed that the administration of 2 doses of IP ghrelin (20 μg/kg total) right before and after severe WD TBI preserved intestinal barrier integrity, restored villous architecture and reduced ileal levels of TNF-α [171].They also demonstrated that the beneficial effects of vagus nerve stimulation (VNS) following TBI may be mediated through ghrelin [172, 173]. VNS was shown to both increase serum ghrelin levels and decrease serum inflammatory cytokines following TBI. Exogenous ghrelin was found to mimic the response seen to VNS by decreasing circulating TNF-α. Importantly, the protective effects of VNS were abolished when a ghrelin receptor antagonist was administered alongside VNS, highlighting a potential ghrelin-dependent mechanism through which VNS works during TBI. The localization of ghrelin receptors to the dorsal motor nucleus of the vagus in the brain supports this link [174]. Furthermore, the administration of 1 dose of intravenous ghrelin (20 μg/kg) 30 min following severe TBI in male rats improved intestinal motility and preserved ileal mucosal architecture [175]. Aside from its important and well-established anti-inflammatory role, ghrelin has been shown to attenuate intestinal barrier dysfunction following intracerebral hemorrhage (ICH) in mice [176]. In a male mouse model of ICH, 2 doses of IP ghrelin (20 μg total) markedly reduced ileal mucosal injury at both histological and ultrastructural levels. Ghrelin also reduced the increase in intestinal permeability commonly seen following ICH by upregulating intestinal tight junction-related proteins Zonula occludens-1 (ZO-1) and claudin-5 [176]. The activation of the mTOR has also been implicated in the gastro-protective mechanisms of ghrelin. Zhang et al. found that ghrelin increased the phosphorylation of mTOR following superior mesenteric artery (SMA) occlusion [177]. This finding did not occur when the specific ghrelin antagonist, growth hormone-releasing peptide 6, was co-administered. Ghrelin’s ability to activate mTOR was ultimately correlated with findings of attenuated organ injury and increased survival in mice that received ghrelin treatment alongside SMA occlusion [177]. There are several mechanisms through which ghrelin can ameliorate both brain and gut injury following an insult. The lack of a consistent mechanism throughout the highlighted papers supports the hypothesis that ghrelin’s pleiotropy contributes to its beneficial impact.

### Progesterone

Progesterone is a steroid hormone primarily produced by the gonads and the adrenal cortex that regulates uterine function and female reproduction [178]. However, research suggests that progesterone plays a role beyond the female reproductive tract, including the CNS, where it is secreted by neurons and glial cells. Its effects include promoting neurogenesis and myelination and improving learning and memory [179]. The role of progesterone has been extensively described in multiple CNS diseases including TBI, Parkinson’s disease and AD, where it has been labeled as neuroprotective due to its anti-inflammatory properties [180]. The therapeutic benefits of progesterone were explored in TBI [181], stroke [182] and neurodegenerative diseases’ animal models [183], where progesterone receptors are expressed in various CNS cells such as neurons, astrocytes and oligodendrocytes, in addition to Schwann cells in the peripheral nervous system [184]. Studies have shown that early administration of progesterone post-TBI reduces the expression of proinflammatory cytokines [185], repairs BBB [186], promotes myelin and axonal regeneration [187], attenuates oxidative damage of mitochondria [188], and improves cognitive and motor outcomes [189]. Progesterone also stimulates synaptogenesis and neurogenesis by increasing the expression of nerve growth factor and brain-derived neurotrophic factor (BDNF) [190].

Guo et al. demonstrated that subcutaneous (SQ) administration of progesterone (16 mg/kg) decreased brain edema 3 days following TBI in male rats by increasing the expression of AQP4 water channels near the lesion site [186]. Other studies also revealed that progesterone attenuates the neuroinflammatory response following TBI by lowering the activation of the transcription factor NF-κB and the expression of the proinflammatory cytokines TNF-α and IL-1β [185, 191]. Ultimately, this leads to a reduction in the activation of cFos, a transcription factor known to promote apoptosis and inflammation [192, 193]. Consistent with these results, Yao et al. demonstrated that progesterone administration following moderate lateral fluid percussion injury (FPI) in male rats decreased the proapoptotic genes Bad and Bax’s expression and increased the anti-apoptotic gene Bcl-2 [194]. Furthermore, progesterone increases circulating endothelial progenitor cells (CD31 and CD34) which facilitates neural regeneration and vascular remodeling, leading to improved cognitive outcomes [187]. An alternative mechanism by which progesterone exerts its therapeutic effects involves the activation of complement decay-accelerating factor (CD55), a potent inhibitor of the neuroinflammatory cascade [195].

One systematic review found that progesterone administration immediately after experimental brain injury reduced the lesion volume [196], but a more recent systematic review showed that it did not reduce mortality or adverse outcomes after TBI [197]. Due to the promising results of numerous preclinical studies, researchers conducted phase II clinical trials to study the efficacy of progesterone in human subjects following acute TBI. In the first phase II trial in Atlanta, GA named ProTECT, progesterone was shown to decrease mortality on post-injury day 30 [198]. Afterward, a more extended clinical trial was conducted in Hangzhou, China, which demonstrated improved functional outcomes and recovery at 3 months post-injury [199]. Ultimately, two phase III clinical trials (ProTECT III and SyNAPSe) failed to confirm the previous promising pro-regenerative results. Nonetheless, research critics attributed its failure to the reliance on subjective measures and the lack of objective measures to accurately evaluate the outcomes. Issues with medication dosage and the tendency to overvalue false positive results in preclinical trials were also identified [200]. However, a notable oversight in launching the trials was the failure to understand how progesterone affects gut motility [201] and the subsequent temporal changes in the gut’s immune profile [66, 202], which could potentially influence neurological outcomes. Even though Phase III clinical trials were disappointing, progesterone may still be regarded as a crucial targeted pharmacological therapy, especially in the absence of any FDA-approved drug for treating TBIs.

Progesterone’s neuroprotective quality has led researchers to study its role in TBI-related intestinal dysfunction. Chen et al. showed that SQ progesterone (16 mg/kg) administration for 5 days helped preserve ileal mucosal integrity and decrease inflammation following TBI by lowering the proinflammatory cytokines IL-1β and TNF-α, as well as reducing cellular apoptosis [203]. Then, they also discovered that progesterone decreased the activation of transcription factor NF-κB, which was thought to be responsible for its therapeutic effects [204]. Subsequent investigations revealed that the antioxidant transcription factor nuclear factor erythroid 2-related factor 2 (Nrf2) also alleviates intestinal inflammation following TBI by decreasing the activation of NF-κB [205], leading to the possibility that progesterone decreases NF-κB activation by modulating the Nrf2 pathway. In addition, Jin et al. observed an elevation in intestinal permeability following TBI in Nrf2-deficient mice, which consequently led to increased levels of plasma endotoxins. This, in turn, resulted in decreased levels of antioxidant and detoxifying enzymes [206]. Furthermore, it is worth mentioning that Nrf2-deficient mice exhibited increased levels of intercellular adhesion molecule-1 (ICAM-1) expression, while progesterone therapy successfully decreased ICAM-1 expression following TBI [204, 205]. These studies demonstrate the restorative synergistic effects of progesterone and the Nrf2 pathway in preserving mucosal integrity. More studies are necessary to fully elucidate the precise mechanisms underlying the effects of progesterone on the Nrf2 pathway via progesterone receptors. Later, Zhou et al. conducted in vitro experiments which demonstrated that progesterone effectively reduced gut permeability by upregulating the expression of the tight junction occludin [207]. In addition, gut dysfunction is observed in brain disorders like subarachnoid hemorrhage (SAH) and Parkinson’s disease, and the use of progesterone has been shown to alleviate intestinal inflammation, suggesting its protective role in brain-gut axis dysfunction. In a male rat model of SAH, IP administration of progesterone (16 mg/kg) for 5 days reduced the expression of proinflammatory cytokines IL-1β, TNF-α and IL-6 and restored mucosal integrity in the ileum [208]. In a mouse model of Parkinson’s disease, the use of progesterone also demonstrated anti-inflammatory effects in the ileal tissues [209]. In conclusion, these studies demonstrate the synergistic role of progesterone in mitigating brain and intestinal inflammation following TBI.

### ANS modulators

#### Beta-blockers

Catecholamines, namely norepinephrine (NE), exert their effects through the activation of beta-receptors: beta1-3 and alpha-receptors: alpha1-2 [210, 211]. Beta1-3 receptors are found in various organs in the body, where they lead to increased cardiac contractility, increased heart rate, vasodilation, and bronchodilation. They also play a role in regulating lipolysis in white adipose tissue, thermogenesis in brown adipose tissue and mobilization of hematopoietic progenitor cells [212]. It has been shown that TBI patients experience sympathetic hyperactivity, causing a catecholamine surge [213–218]. This surge causes secondary insults, which manifest as posttraumatic hyperthermia [216, 218], increased inflammation and leukocyte migration [219, 220], impaired cerebral homeostasis and perfusion [217], and cardiovascular dysfunction [216], and has been associated with increased mortality [221]. In light of such evidence, the use of beta-blockers to alleviate secondary damage caused by TBI gained interest. Beta-blockers can be selective to one type of receptor, such as metoprolol (beta-1 receptor blocker), or act non-selectively on all beta-receptors, such as propranolol. Some, like labetalol (beta-1 and 2 receptor blocker), can also act on alpha-1 receptors, thereby altering the expected outcome [212].

A study by Lopez et al. subjected male mice to severe TBI via controlled cortical impact and tested the effects of treating them with the non-selective beta-blocker propranolol for two [219] and fourteen days [220]. Propranolol treatment led to decreased penumbral leukocyte migration in a dose-dependent manner and improved weight recovery at both timelines and decreased cerebral edema and albumin leakage at two days. Other preclinical trials revealed decreased p-tau accumulation [222], improved cerebral oxygenation and perfusion [212], restoration of cerebral autoregulation [217], decreased hippocampal necrosis [217], higher neurologic scores [212, 219] and improved behavioral [222], cognitive and memory [223] performances with beta-blocker treatment. Beta-blockers are hypothesized to act by (a) providing a neuroprotective environment by increasing protective proteins, such as heat shock protein 70 (HSP-70) and downregulating ubiquitin carboxyl-terminal hydrolase L1 (UCHL-1), which is involved in oxidative damage [223]; (b) decreasing calcium entry into the cell, which decreases apoptosis [223]; (c) modulating microglial function and synaptic plasticity [224]; and (d) inhibiting pro-inflammatory IL-6 signaling [217].

The success seen with beta-blocker treatment in preclinical trials translated well into clinical studies. Observational studies showed a significant decrease in mortality in TBI patients treated with beta-blockers, particularly propranolol, compared to those administered placebo [225] and better long-term neurologic functioning [215]. Results were associated with increased length of hospital stay with inconsistent results on length of ICU stay [214, 216, 225]. The adverse effects of using beta-blockers included a longer time on the ventilator and increased infection rates [214, 216]; however, both beta-blocker and placebo groups experienced similar rates of cardiopulmonary adverse events such as hypotension, bradycardia, heart blocks, arrest, and bronchospasms [215]. Asmar et al. demonstrated that beta-blockers, particularly propranolol, play a role in alleviating the dysregulation in body temperature after TBI by significantly decreasing the number of hyperthermic episodes, lowering the median temperature, and spacing out the episodes. The results were more pronounced in patients with severe head injury, and these patients further showed decreased lengths of ICU stay and higher Glasgow Coma Scale scores at discharge [218]. In a prospective randomized controlled trial, Khalili et al. showed that treating patients with severe isolated TBI with propranolol resulted in decreased mortality and improved functional outcomes at 6 months compared to placebo (Level II evidence) [226]. The Eastern Association for the Surgery of Trauma (EAST) recommends the use of beta-blockers in adult TBI patients with no contraindications and only in an ICU setting where cardiovascular side effects, primarily hypotension, and bradycardia, can be monitored and treated promptly [213].

Evidence shows that TBI is associated with increased intestinal permeability, which correlates with increased mortality, that is significantly reduced with beta-blocker treatment [221, 227]. Lang et al. also showed that male rats subjected to TBI had increased epinephrine levels, higher intestinal TNF-a levels, decreased ZO-1 protein expression, which forms tight junction in the intestine, and worse mucosal damage on ileum histopathology compared to sham rats. All these changes were reversed significantly when the rats were treated with 1 dose of IP labetalol (beta-1 and 2 receptor blocker) right after TBI [227]. Gut dysfunction seen in TBI can also induce changes in the brain via NE signaling that can further exacerbate gut dysfunction as NE plays a bidirectional role in the brain-gut axis. For instance, rat models show that gastric distension induces a signification increase in NE in the ventral bed nucleus of the stria terminalis (vBNST), which is known to be involved in response to stress. In turn agonism of beta-receptors in the vBNST leads to a significant decrease in gastric emptying and small intestinal transit, which is reversed with the administration of beta-blockers [228].

#### Alpha-agonists/antagonists

In the setting of the brain-gut axis, alpha-adrenergic receptor modulation remains relatively unstudied. Despite the lack of bi-directional data on the therapeutic action of alpha receptor modulators, their action on the brain following TBI is of significant interest in both translational and clinical research settings [229, 230]. In male rats, the alpha-2 adrenergic agonist mafedine promotes the restoration of interhemispheric connectivity in remote brain regions and intrahemispheric connection within the unaffected hemisphere post-TBI day 7 [231]. The authors also found that mafedine improved cortical response to photic and somatosensory stimulation [231]. Alongside alpha-2 agonists, the role of alpha-1 antagonists is also being actively explored in the setting of TBI. Kobori et al. have shown that IP administration of the alpha-1 antagonist (2-[b-(4-Hydroxyphenyl)ethyl]aminomethyl-1-tetralone hydrochloride (HEAT)) (0.1 mg/kg) improved working memory in male Sprague Dawley rats post-TBI day 14 [232]. Such studies suggest that modulation of alpha adrenoceptors and, by extension, norepinephrine signaling may benefit TBI patients. These findings add to existing clinical data from the COMA-TBI study showing that elevated catecholamines are associated with unfavorable outcomes following isolated moderate to severe TBI [73, 233]. Importantly the COMA-TBI study highlights the differences in the predictive use of both epinephrine (Epi) and norepinephrine (NE), with admission levels Epi associated with higher rates of unfavorable outcomes and mortality and changes in NE associated with higher mortality risks. The differences in the predictive value of each of these catecholamines may well be due to their affinities for adrenoceptor sub-types.

In the clinical setting, the role of alpha-2 agonists has been investigated extensively in the setting of severe TBI with the hopes that these agents may attenuate sympathetic hyperactivity following brain injury [234]. Clonidine (alpha-2 agonist), in combination with propranolol, has been studied in patients with severe TBI as it reduces levels of circulating catecholamines and decreases levels of cerebral vasoconstriction without altering cerebral blood flow [235]. To date, several studies, including the “DASH After TBI Study” [236] and the Nordness et al. [237] pilot study have found that while adrenergic blockade with propranolol and clonidine is safe and feasible, the drugs do not alter ventilator-free day numbers in patients with severe TBI.

### Statins

Since their discovery in the early 1970s, statins have become the mainstay treatment for patients with high cholesterol levels [238]. As research advanced, the use of statins expanded due to their anti-inflammatory properties where they have been shown to be effective in the management of coronary artery disease [239], arthritis [240], brain injuries [241] and intestinal inflammation [242]. The protective role of statins in the CNS following brain injuries has been widely described [243–245]. Statins have been shown to attenuate neurodegeneration by decreasing neuronal death, apoptosis, microglial activation and astrogliosis [246]. Statins also promote neurogenesis, synaptogenesis and angiogenesis in the brain, particularly in the boundary zone of the lesion and the hippocampus [247]. Studies have shown that statin use after TBI is associated with decreased risk of death and improved function and capacity at 12 months post-injury [248]. In a randomized double-blind clinical trial, statins improved functional recovery at 3 months post injury, although they had no effect on brain contusion volume [249]. Rat animal models also showed enhanced spatial memory function with atorvastatin treatment following TBI [250, 251]. A systematic review by Sultan et al. suggested that statins display a neuroprotective role, particularly in improving cognitive outcomes and reducing the risk of dementia [243]. Statins were also shown to reduce beta-amyloid peptide levels post TBI, which may play a role in the improvement in cognitive outcomes and decreasing the risk of AD in TBI patients [252].

The anti-inflammatory effects of statins are numerous, such as reduction in the expression of proinflammatory markers TNFα and IL-1β, as well as a decrease in the activation of microglia and astrocytes, as evidenced by a decrease in the expression of cluster of differentiation 68 (CD68) and glial fibrillary acidic protein (GFAP), respectively [253, 254]. Statins reduce astrogliosis by modulating caveolin-1 expression and epidermal growth factor receptor in astrocyte lipid rafts [255]. Statins also upregulate the expression of vascular endothelial growth factor (VEGF) and BDNF in the dentate gyrus of the hippocampus through Akt-dependent signaling pathways, upregulating the expression of GSK-3beta and cAMP response element-binding proteins, ultimately increasing cellular proliferation and enhancing neurogenesis [256]. In addition, daily oral simvastatin administration (1 mg/kg/day) reduces neuronal apoptosis following TBI in male rats through increased Akt phosphorylation and activation of its downstream targets, such as Forkhead transcription factor 1, inhibitory-kappaB, and endothelial nitric oxide synthase, while attenuating the activation of caspase-3. All these effects lead to improved neuronal survival and function [257]. Furthermore, statins decrease brain edema following injury by reducing BBB permeability due to a decrease in ICAM-1 and neutrophil infiltration [258]. Taken together, these findings suggest that statins play a beneficial role in attenuating the neuroinflammatory response post-TBI and improving outcomes.

The protective role of statins along the brain-gut axis is emerging. First, it is significant to note that cluster of differentiation 40 (CD40), a transmembrane receptor of the tumor necrosis factor receptor family, is strongly implicated in intestinal inflammation in various diseases such as TBI, IRI and IBD [259]. In a rat model of TBI, Hu et al. demonstrated that the expression of CD40 increases in the jejunum which is positively correlated with an increase in the activity of NF-κB and levels of TNF-α, vascular cell adhesion molecule-1 (VCAM-1) and ICAM-1 [260]. Ultimately, studies revealed that one dose of IP (30 mg/kg) rosuvastatin right after TBI attenuated jejunal injury by reducing the expression of TNF-α and IL-1β and blocking the CD40/NF-κB pathway. Statins also alleviated TBI-induced intestinal morphometric alterations by preserving normal mucosal architecture and maintaining villous integrity [261]. Furthermore, in vivo (experimental murine colitis model) and in vitro (intestinal epithelial cells) studies both reconfirmed the anti-inflammatory properties of statins by blocking the activity of NF-κB, inhibiting the phosphorylation of IκB and eventually lowering the expression of TNF-α [262]. Statins have also been shown to decrease CD40 expression in human vascular cells [263]. In addition, Ozacmac et al. revealed that daily PO atorvastatin (10 mg/kg) 3 days before IRI promoted gut motility and ileal contractility possibly due to a decrease in oxidative stress, neutrophil accumulation and tissue malondialdehyde expression, as well as an increase in reduced glutathione levels [264]. An alternative mechanism in which statins exert their protective effects in the gut is by modulating the gut microbiome. A growing body of evidence suggests that TBI alters the composition of the gut microbiome and promotes pathogenic bacteria over commensal bacteria mainly due to intestinal dysmotility and increased paracellular permeability, therefore, aggravating disease progression [4]. In turn, the vagus nerve can reciprocally affect the CNS by sensing the microbial products through its afferent fibers [99]. This leads to a deleterious cycle between the injured brain and the dysbiotic gut. One possible way to halt this inflammatory cycle is through the use of statin therapy. Statins have been shown to promote healthy gut flora and ameliorate gut dysbiosis [265]. Studies have also shown that statins demonstrate anti-inflammatory effects in IBD and are linked to a decreased requirement of steroids [242, 266]. The potential use of these drugs is promising and interesting. More studies are encouraged to better elucidate how statin therapy regulates the brain-gut axis in the context of TBI.

### Microbiome modulators

#### Antibiotics

Recent investigations indicate that gut microbial dysbiosis impacts intestinal function not only in diseases like IBS and IBD but also in neurologic conditions such as TBI and strokes [267, 268]. TBI promotes the presence of pathogenic bacteria over commensal bacteria, leading to heightened intestinal inflammation, increased barrier permeability and dysmotility [93, 268]. To gain a better understanding of alterations in the microbiome and their implications on brain inflammation after TBI, studies have utilized various antibiotic combinations to deplete the microbiome, in addition to the administration of beneficial probiotic strains to promote a healthy intestinal flora. Many animal studies initially utilized germ-free (GF) mice to elucidate relation between microbial dysbiosis and the host’s physiologic response [71, 269]. A study by Simon et al. demonstrated that administering a combination of antibiotics (1 g/L of ampicillin, metronidazole, neomycin and vancomycin) two weeks before TBI in male mice improved intestinal barrier permeability, as evidenced by increased ZO-1 staining along the cecum epithelium. This resulted in decreased microglial activation, increased hippocampal neuronal density, reduced lesion volume, and improved learning outcomes [269]. Conversely, another study by Celorrio et al., using the same antibiotic regimen for two weeks before injury, showed worsened neuronal loss in the hippocampus, triggering a more pronounced fear response in adult male mice [202]. The opposing outcomes may be attributed to various factors, including differences in mice intestinal flora in distinct animal facilities, variations in diet impacting the host microbiome, and differences in injury severity and time points of behavioral analysis [270, 271]. Celorrio et al.’s study also revealed the intricate interplay between the gut microbiome and the immune system. Antibiotic administration for 1 week after TBI reduced the infiltration of peripheral monocytes and T-lymphocytes into the brain while increasing microglial inflammatory markers 3 days after injury. The heightened expression of proinflammatory surface markers (TLR4 and MHCII) by microglia, along with alterations in their morphology toward an amoeboid shape, correlated with decreased neurogenesis in the dentate gyrus and increased neuronal degeneration in the CA3 region of the hippocampus [71, 202]. Benakis et al. also demonstrated that alterations in the intestinal flora of male mice induced by a two-week course of amoxicillin-clavulanic acid (1 g/L) resulted in reduced ischemic brain injury. This reduction was attributed to an increase in intestinal regulatory T-cells and a decrease in the infiltration of IL-17 + γδ T cells into the brain, observed 16 h post-injury [70]. Targeting the gut-microbiota-immune axis offers a promising approach to improve recovery following TBI.

#### Probiotics

In an effort to better understand microbiome changes, the administration of probiotics has been widely experimented in TBI models. Probiotics regulate microbial populations in the gut lumen by favoring the colonization of beneficial bacteria and reducing pathogenic populations [272]. They enhance epithelial cell differentiation and proliferation and maintains epithelial barrier integrity, thereby limiting the translocation of harmful bacteria into the intestinal parenchyma [273]. A study by Ma et al. showed that the supplementation of probiotics, specifically LA for 7 days after weight-drop TBI, has protective effects on the ileum after TBI. This was revealed by improvements in intestinal barrier function and the gut’s absorptive capacity for nutrients and electrolytes [274, 275]. The effects of LA also included improvements in ileal villous architecture and enhanced ileal motility through the restoration of the gut’s pacemaker cells, known as the interstitial cells of Cajal (ICC), by increasing levels of myosin light chain kinase protein and promoting smooth muscle contraction [276]. *Lactobacillus reuteri* was also utilized in clinical trials involving military veterans with mild TBI. Results demonstrated significant reductions in serum inflammatory C-reactive protein levels in patients taking *Lactobacillus reuteri* daily for 8 weeks compared to the placebo group [277]. Furthermore, a systemic review and meta-analysis conducted by Du et al. showed that early enteral nutrition with probiotics after TBI reduced mortality and decreased GI complications such as constipation, abdominal distention, reflux and stress ulcers [278].

Probiotics also stimulate the release of SCFAs, such as acetate, butyrate, and propionate, into the peripheral circulation which can ultimately cross the BBB and ameliorate brain inflammation by decreasing the expression of proinflammatory cytokines and promoting neurogenesis [279–281]. A study by Li et al. demonstrated the neuroprotective effects of *Clostridium butyricum* (Cb), a butyrate-producing probiotic, after TBI via the brain-gut axis by increasing the levels of GLP-1 and glucagon-like peptide 1 receptor in the colon. Intragastric Cb supplementation (10^9^ CFU/ml), once daily for 2 weeks pre and post-TBI, improved neurological outcomes in male mice by attenuating neurodegeneration, decreasing apoptosis and enhancing BBB integrity [282]. In parallel, the administration of the probiotic *Lactobacillus acidophilus (LA)* (1 × 10^10^ CFU) for 7 days after weight-drop TBI shifts the microbiome towards a healthier profile in male mice. LA improved neurological outcomes by decreasing the activation of astrocytes and microglia, and reduced brain edema by preserving the BBB integrity. LA supplementation also decreased the expression of gram negative bacteria receptors in the brain, mainly TLR4 [275]. Last, a randomized clinical trial showed that the daily administration of a combination of probiotics (*Bifidobacterium longum, Lactobacillus bulgaricus, Streptococcus thermophilus*) for 21 days after severe TBI attenuated systemic inflammation by lowering the levels of IL-4 and IL-10 and reducing the risk of nosocomial infections [283].These studies emphasize the link between the host gut microbiome and immune responses in the brain following TBI. The utilization of antibiotics/probiotics holds substantial therapeutic potential in TBI management, with further studies warranted to better elucidate their effects on brain inflammation and behavioral outcomes.

#### Microbial metabolites

Studies have also revealed that TBI induces alterations in gut microbial metabolites, including SCFAs and bile acids. Among these metabolites, SCFAs such as butyrate, propionate and acetate play a crucial role [284]. They serve as alternative energy sources for the injured brain and help regulate mitochondrial homeostasis [285]. A study by Opeyemi et al. demonstrated a significant decrease in fecal SCFAs concentrations 24 h and 28 days post-TBI in adult male mice. This decrease correlated with worse learning outcomes, while the administration of SCFAs improved spatial learning in these mice [286]. Furthermore, the role of bile acids has been studied in various neurological diseases, such as intracranial hemorrhage and ischemic stroke [287, 288]. The administration of tauroursodeoxycholic acid has been shown to improve neurologic function and decrease infarct size two and seven days after reperfusion [289]. In the context of TBI, You et al. demonstrated decreased levels of bile acids in serum and fecal samples one day after TBI in adult male mice [268]. Moreover, Zhu et al. conducted a prospective study which showed decreased levels of plasma bile acids in TBI patients [290]. Therefore, supplementation of microbial metabolites byproducts such as bile acids and SCFAs present promising therapies for mitigating neuroinflammation post-TBI.

#### Mycobiome

In addition to the dominant bacterial microbiome, the gut mycobiome (fungal microbiome) emerges as another crucial therapeutic target, regulating brain-gut homeostasis. Imbalances in the mycobiome have been observed in various intestinal diseases including IBS [291], IBD [292] and colorectal cancers [293]. Visceral hypersensitivity in IBS has been associated with increased fungal dysbiosis [294]. While their role is still understudied in traumatic brain injury, a recent prospective observational cohort study conducted by Park et al. revealed dysregulated mycobiome balance in critically ill trauma and sepsis for up to two to three weeks after intensive care unit hospitalizations. Dominance in *Candida* spp population and reduction in commensal fungal species correlated with heightened vulnerability to infections [295]. Interestingly, another study by Hunag et al. demonstrated that changes in gut mycobial abundance in mice three days post-TBI were linked to dysfunctional regulation of N^6^-methyadensoine (m^6^A), which is crucial in posttranscriptional modification of eukaryotic mRNA [296]. Given the intricate relationship between bacterial and fungal microbiomes, more studies are warranted to better understand the role of fungemia, particularly, candidemia, in TBI patients, and how modulating the mycobiome can modulate neurologic outcomes.

#### Current limitations

Human and animal studies are pivotal in investigating microbiome alterations and their impact on neurological outcomes following TBI [297, 298]. Various methodologies are utilized to detect these changes, including the administration of antibiotics [299], probiotics [283], SCFAs [300], and the use of fecal microbiome transplant [301]. Studies on the impact of antibiotics are particularly crucial, given that some patients with open head injuries and polytrauma necessitate antibiotic administration [302, 303], yet the neurological consequences of such interventions remain poorly understood. Very few clinical studies have examined the impact of antibiotics on TBI outcomes and there are currently no general guidelines on the administration of antibiotics [299]. In the context of TBI-induced gut microbial dysbiosis, there is a need to develop well-controlled clinical trials to explore the effects of antibiotic administration on changes in infection rates and neurological outcomes over the long-term.

A primary challenge in microbiota research in TBI originates from the widespread variability in sample collection and processing methods, including targeted sequencing and metagenomic sequencing [304]. Moreover, the heterogeneity in individual’s dietary patterns and pre-existing microbiome diversity present additional challenges in detecting microbiome variations [305]. Although preclinical animal models offer controlled experimental conditions such as standardized environments, their findings pose challenges in translational efforts [306]. Another significant aspect is the time required for probiotics to exert their effects on neurocognitive outcomes, potentially spanning years [307]. Thus, well-designed long-term clinical trials are necessary to fully comprehend the impact of microbiome in TBI. Addressing these limitations is essential for developing effective therapeutic interventions.

Tables 1 and 2 present a summary of various anti-inflammatory agents that attenuate brain and gut inflammation following TBI.

**Table 1:** Summarized findings of the impact of various pharmacotherapies on TBI-induced neuroinflammation

Therapy	Intervention	Sex	Species	Model	Timepoints	Impact on Brain Inflammation
Serotonin	IP SSRIs (fluoxetine) 5 mg/kg, single dose immediately after injury	N/A	Mice	Single unilateral, severe TBI (CCI)	7 days post-TBI	Improved motor recovery and coordination on rota-rod [117]
	SNRIs (Milnacipran) 30 mg/kg/day via IP osmotic minipumps until sacrifice	Male	Rats	Single unilateral, moderate TBI (CCI)	5 weeks post-TBI	Enhancement of serotonergic tone in the medial prefrontal cortex and improvement of attentional set-shifting task performance [111]
	PO SSRIs (sertraline) 50 mg daily for 24 weeks to patients with history of TBI	Male	Humans	Moderate-Severe TBI	24 weeks post-treatment	Significant improvement in mood and depressive symptoms [119]
Ghrelin	2 doses of IP ghrelin (20 μg total), one hour before and right after TBI	Male	Mice	Single unilateral TBI (WD)	6 h post-TBI	Prevents BBB disruption by decreasing vascular permeability and AQP-4 expression in the injured area of the cortex [159]
	1 dose of IV ghrelin (20 μg/kg) 30 min after injury	N/A	Rats	Single unilateral TBI (CCI)	24, 48 and 72 h post-TBI	Reduces brain inflammation and improves neurological severity score by decreasing basic fibroblast growth factor (bFGF) in the injured cortex [162]
	3 doses of IP des-acylated ghrelin (1 mg/kg) on same day after stroke	Male	Mice	Transient middle cerebral artery occlusion (tMCAo)	24 h post-stroke	Reduces infarct size, decreases apoptosis and maintain BBB integrity by upregulating tight junction proteins occludin and claudin-5 in the ischemic cerebral hemisphere [167]
Progesterone	3 doses of SQ Progesterone (16 mg/kg) 6, 24 and 48 h post-TBI	Male	Rats	Bilateral TBI (CCI)	72 h post-TBI	Reduces brain edema by decreasing the expression of peri-contusion AQP-4 channels in the injured cortex [186]
	IP Progesterone (8 mg/kg) and Allopregnanolone (4 mg/kg) for 5 days post-TBI	Male	Rats	Bilateral, severe TBI (CCI)	6 days post-TBI	Reduces expression of proinflammatory cytokines IL-1B and TNF-a in the frontal lobe [185]
	SQ Progesterone (16 mg/kg) daily for 14 days post-TBI	Male	Rats	Single unilateral moderate TBI (FPI)	2 weeks post-TBI	Improves neurological outcomes on MWM test and induces vascular remodeling in the injured parietal lobe by increasing circulating endothelial progenitor cells [187]
Beta-blockers	1 dose of IV propranolol (1 mg/kg) 1 h post-TBI	Male and Female	Pigs	Single moderate TBI (FPI)	4 h post-TBI	Decreases hippocampal neuronal death by downregulating IL-6 expression [217]
	IP propranolol (1–4 mg/kg) daily for 2 and 14 days post-TBI	Male	Mice	Single severe TBI (CCI)	2 and 14 days post-TBI	Reduces edema and preserves cerebral BBB permeability in a dose-dependent manner [219, 220]
	IP propranolol (4 mg/kg) daily for 7 days post-TBI	Male	Mice	Single unilateral moderate TBI (CCI)	7 days post-TBI	Improves memory, learning and cognition by reducing cell death in the hippocampal-CA1 area [223]
Statins	PO Simvastatin (1 mg/kg/day) daily until sacrifice	Male	Rats	Single TBI (CCI)	1, 3, 7, 14, and 35 days post-TBI	Downregulates the expression of inflammatory cytokines and decreases the activation of astrocytes and microglia around the lesion boundary zone [253]
	PO Simvastatin (1 mg/kg/day) daily until sacrifice	Male	Rats	Single unilateral TBI (CCI)	1, 3, 7, 14, and 35 days post-TBI	Improves spatial learning and promotes neurogenesis by increasing VEGF and BDNF expression in the ipsilateral hippocampus through Akt-dependent signaling [256, 257]
	2 doses of PO simvastatin (37.5 mg/kg) 1 and 6 h post-TBI	Male	Rats	Single unilateral moderate TBI (FPI)	24 h post-TBI	Reduced brain edema by preserving BBB integrity through upregulating claudin-5 [258]
Antibiotics/probiotics	PO ampicillin, metronidazole, vancomycin, neomycin (1g/L) daily for 2 weeks pre-TBI	Male	Mice	Single unilateral TBI (CCI)	3 days post-TBI	Increases hippocampal neuronal density and reduces lesion volume [269]
	Intragastric gavage of Clostridium butyricum (10^9^ CFU/ml) once daily for 2 weeks pre and post-TBI	Male	Mice	Single TBI (WD)	2 weeks post-TBI	Improves neurological dysfunction, decreases brain edema and reduces neuronal degeneration around the lesion site [282]
	PO gavage lactobacillus acidophilus (1 × 10^10^ CFU) one daily until sacrificed	Male	Mice	Single unilateral TBI (WD)	1,3 and 7 days post-TBI	Decreases the activation of astrocytes and microglia in the injured cortex and reduces brain edema by preserving the BBB integrity [275]

CCI, controlled cortical impact; WD,  weight drop; FPI, fluid percussion injury; TBI,  traumatic brain injury

**Table 2:** Summarized findings of the impact of various pharmacotherapies on TBI-induced gut inflammation

Therapy	Intervention	Sex	Species	Model	Timepoints	Impact on gut inflammation
Serotonin	SSRIs (IP fluoxetine) 5 mg/kg, single dose immediately after injury	N/A	Mice	Single unilateral, severe TBI (CCI)	4 days post-TBI	Preserves colonic barrier integrity by reducing FITC-dextran permeability [117]
Ghrelin	2 doses of IP ghrelin (20 μg total), one hour before and right after TBI	Male	Mice	Single unilateral, severe TBI (WD)	6 h post-TBI	Maintains ileal barrier integrity and architecture and decreases TNF-α levels [171]
	1 dose of IV ghrelin (20 μg/kg) 30 min after TBI	Male	Rats	Single unilateral severe TBI (CCI)	1–3 days post-TBI	Improves intestinal motility and protects ileum mucosal epithelium [175]
	2 doses of IP ghrelin (20 μg total) right after ICH	Male	Mice	Intracerebral hemorrhage (ICH)	1 day post ICH	Reduces ileal permeability by upregulating tight junctions ZO-1 and claudin-5 [176]
Progesterone	SQ progesterone (16 mg/kg) daily for 5 days post-TBI	Male	Rats	Single unilateral TBI (WD)	5 days post-TBI	Downregulates the expressions of ileum TNF-α, IL-1β, and ICAM1 and reduces mucosal apoptosis [203]
	SQ progesterone (16 mg/kg) daily for 5 days post-TBI	Male	Rats	Single unilateral TBI (WD)	5 days post-TBI	Reduces ileum NF-κB activation and proinflammatory cytokines expression [204]
	IP progesterone (16 mg/kg) daily for 5 days post-SAH	Male	Rats	Subarachnoid hemorrhage (SAH)	5 days post-SAH	Restores ileum mucosal integrity and reduces proinflammatory cytokines IL-1β, TNF-α and IL-6 [208]
Beta-blockers	1 dose of IP labetalol (30 mg/kg) right after TBI	Male	Rats	Single unilateral TBI (WD)	3, 6, and 12 h post-TBI	Decreases hyperactivity of adrenergic tone, reduces intestinal TNF-α levels, and prevents an increase in ileum permeability [227]
	PO labetalol (62.5 μM in 1 M sucrose) after TBI	Male and Female	Drosophila	Closed head TBI using high-impact trauma device	24 h post-TBI	Reduces intestinal permeability and early mortality [221]
Statins	One dose of IP rosuvastatin (30 mg/kg) right after TBI	Male	Rats	Single unilateral TBI (WD)	24 h post-TBI	Downregulates jejunal TNF-α and IL-1β levels and enhances villous histology by blocking the CD40/NF-κB pathway [261]
	PO atorvastatin (10 mg/kg) daily 3 days before inducing ischemia	Male	Rats	Intestinal ischemia reperfusion injury (IRI)	3 h post-reperfusion	Promotes ileum motility possibly through reducing oxidative stress and increasing glutathione levels [264]
Antibiotics/probiotics	PO ampicillin, metronidazole, vancomycin, neomycin (1g/L) daily for 2 weeks pre-TBI	Male	Mice	Single unilateral TBI (CCI)	3 days post-TBI	Improves intestinal permeability by increasing epithelial ZO1 expression in the cecum [269]
	PO gavage lactobacillus acidophilus (1 × 10^10^ CFU) one daily until sacrificed	Male	Mice	Single unilateral TBI (WD)	1,3 and 7 days post-TBI	Reduces ileum inflammation, maintains barrier integrity, and promotes gut motility through PKC/MLCK/MLC signaling pathway [276]
	PO gavage lactobacillus acidophilus (1 × 10^10^ CFU) one daily until sacrificed	Male	Mice	Single unilateral TBI (WD)	1,3 and 7 days post-TBI	Improves ileum barrier function and gut’s absorptive capacity for nutrients and electrolytes [275]

CCI, controlled cortical impact; WD, weight drop; FPI, fluid percussion injury; TBI, traumatic brain injury; N/A, not available

## Brain-gut axis dysfunction in military veterans

Military veterans provide a unique human model for studying the impact of traumatic brain injuries on the gut in a realistic environment [308]. Following military occupational training involving blast exposure, a significant increase in intestinal permeability and evidence of gut bacterial translocation into the circulation were observed 1 to 16 h later, accompanied by a stepwise increase in alpha microbial diversity and elevated levels of intestinal permeability protein biomarkers such as zonulin and occludin-3 [309]. Additionally, another study involving 26 male Operation Enduring Freedom/Operation Iraqi Freedom/Operation New Dawn (OEF/OEI/OND) veterans revealed that differences in gut microbial composition, specifically Verrucomicrobiota, correlated with psychiatric and cognitive symptoms, including fatigue, depressive symptoms, and PTSD severity, as well as difficulties with attention, executive function, learning and memory [310]. These findings emphasize the intricate relationship between gut microbiota and mental health in veterans. Furthermore, research on Gulf War veterans indicated that a higher abundance of phyla Bacteroidetes, Actinobacteria, Euryarchaeota, and Proteobacteria, as well as higher abundances of the families Bacteroidaceae, Erysipelotrichaceae, and Bifidobacteriaceae, correlated with increased GI symptoms severity and higher levels of the inflammatory cytokine TNF-α, alongside reports of chronic pain, fatigue and sleep difficulties [311]. The Veterans in the US Veteran Microbiome Project (US-VMP) demonstrated a significant association between microbiome composition, episodes of gastroenteritis and symptoms of severe depression [312]. Additionally, an RCT conducted on OEF/OEI/OND veterans revealed that daily administration of Lactobacilli Reuteri for 8 weeks resulted in a decrease in plasma CRP levels compared to untreated groups, indicating the potential of microbiome-targeted interventions in mitigating post-TBI inflammation [277]. Conversely, some studies on US military veterans and US Marines did not find significant differences in gut microbial diversity and permeability after moderate/severe TBI [313, 314]. These conflicting findings underscore the complexity of studying the microbiome in real-world settings. Numerous limitations exist in such studies due to challenges in identifying true control participants, variations in the timing of stool collection, and differences in diet regimens and medications, which significantly impact the microbiome [313]. Efforts to address these limitations and refine study methodologies are crucial for advancing our understanding of the brain-gut axis in the context of TBI in military populations.

## Why do TBI treatments keep failing?

Despite extensive TBI research and significant investment in various therapies, there remains a lack of effective treatments to attenuate disease progression [315]. Many clinical trials have failed to replicate the anti-inflammatory effects observed in preclinical animal models due to several challenges [316]. First, TBI is inherently heterogenous and triggers complex pathogenic pathways, rendering it a highly intricate disease to manage [317]. In basic science, most of the research is focused on specific cellular processes in the brain, often missing the broader picture of the disease [316]. Additionally, each model utilizes different injury paradigms and anatomical locations to induce injury. It is essential to recognize that focal contusion, concussion, and blast injuries elicit distinct inflammatory pathways in response to injury [318]. The tendency to extrapolate inflammatory models from focal contusion to concussion and blasts presents a significant issue due to each injury type’s unique inflammatory profiles [319, 320].

Another key challenge in TBI research is the overrepresentation of males in both clinical trials and preclinical models [321]. Sex differences in TBI responses are evident, with males showing better recovery in clinical trials and females exhibiting superior outcomes in preclinical studies [322]. Emerging evidence suggests the crucial role of sex in brain-gut homeostasis by modulating the gut microbiota and immune system activation [323, 324]. Animal studies reveal notable differences in gut microbiome composition between males and females, with females demonstrating greater diversity [325, 326]. Although the impact of sex on the microbiome in TBI is underexplored, studies in other diseases suggest that females are more susceptible to autoimmune diseases due to microbial alterations [327, 328]. Similarly, the higher prevalence of IBS in women may be linked to microbiome changes driven by estrogen-mediated responses or compromised gut barrier function [329]. To comprehensively understand the sex’s impact on brain-gut axis, clinical trials and animal models with more female representation are warranted.

The complexity of immune response post-TBI presents another challenge [330]. The evolution of immune cell activation over time involves both beneficial and detrimental components, making attempts to target specific immune pathways often unsuccessful [331]. Furthermore, the existing classification of M1/M2 microglia based on in vitro cell culturing excludes the diverse array of cytokines and cellular signals encountered by these cells in vivo, particularly in a complex disease like TBI [332]. A shift away from M1 and M2 classification towards incorporating transcriptomic and proteomic profiling for better analysis is needed [333]. Additionally, inflammation in TBI is not confined to the CNS; there is also global systemic immune dysfunction that remains understudied [57]. The gut, which harbors the largest immune reservoir, exhibits significant alterations in macrophage and T-cell immune profile, necessitating a better understanding of interactions between intestinal immunity and brain immune cells [4, 66].

Most drugs lose efficacy with increasing intervals between injury time and initiation of treatment [334]. Moreover, certain treatments, such as probiotics, require prolonged administration periods, potentially spanning years before cognitive improvements are observed [307]. The therapeutic time window in treatment of TBI remains a significant focus of investigation to advance the development of TBI therapies and successfully translate from preclinical to clinical settings [335]. While animal models often receive therapy immediately after injury, clinical trials at specialized trauma centers typically enroll patients 4–7 h after moderate to severe TBI [336]. Additionally, individuals with mild injuries may delay treatment until they are symptomatic, which may occur years after injury [337, 338]. The significance of the therapeutic time window was underscored by the failure of progesterone treatment in phase III clinical trials PROTECT and SYNAPSE [339]. Both trials recruited patients within 4 and 7 h after TBI, respectively, failing to replicate the benefits observed in preclinical animal models where progesterone was administered within an hour of injury [340]. These clinical trial failures highlight the importance of therapeutic window in initiating treatment. Preclinical models are encouraged to investigate drug efficacy with progressively delayed dosing post-injury to simulate real-life settings.

In addition to treatment timing, understanding the optimal duration of treatment is essential for elucidating long-term outcomes [341]. While some drugs, such as beta-blockers, have demonstrated significant improvements with short-term administration, even as brief as two days or two weeks, others, like probiotics, may require prolonged treatment periods to exert neuroprotective effects, especially for chronic conditions like TBI [219, 220, 277, 342]. A comprehensive understanding of the temporal and spatial dynamics of immune, hormonal and neural pathways within the brain-gut axis is imperative for evaluating the effects of chronic treatment regimens post-TBI [343]. Current animal studies predominantly focus on acute changes, missing potential chronic alterations that may influence drug efficacy and subsequent neurologic outcomes [15]. Persistent microbiome alterations observed for years post-TBI emphasize the potential necessity for extended treatment durations to mitigate brain injury consequences [89]. Studying the therapeutic window for treatment, the duration of treatments, and their impact on brain-gut homeostasis is crucial for the development of successful TBI therapies [334].

In clinical trials, numerous challenges are encountered, including the nonuniform population of patients with comorbid diseases and ethical considerations for randomization [344]. Additionally, the classification of mild-moderate-severe TBI is overly broad and fails to account for radiological and clinical findings, making it subjective [345]. Improved classifications systems are necessary to address this issue. The recent FDA approval for Abbott’s i-STAT TBI cartridge, designed to identify blood biomarkers such as GFAP and UCH-L_1_ within just 15 min at the patient’s bedside, promises more objective results to stratify injury severity and tailor management away from the conventional mild-moderate-severe classification [346]. Last, the reliance on monotherapy to treat such a complicated disease compound the challenges [347]. There should be greater emphasis on targeting various pathways in the brain and gut at the same time to effectively manage this complex disease.

## Conclusion

In conclusion, we present the first comprehensive review of various pharmacotherapies targeting inflammation along the brain-gut axis in TBI, which include hormones such as serotonin, ghrelin, and progesterone, alongside ANS modulators like beta-blockers and alpha-adrenergic agonists, antilipidemic agents such as statins, and intestinal flora modulators such as probiotics and antibiotics (summarized in Tables 1 and 2). They enhance gut function by mitigating inflammation, preserving intestinal barrier integrity, promoting motility and favoring a healthy microbiome. Importantly, these pharmacotherapies also impact neurological outcomes directly by crossing the BBB or indirectly by modulating immune, hormonal and neural pathways.

With the gut serving as the largest immune reservoir, housing the body’s second “little brain”-the ENS [348], and hosting a diverse microbiome, it emerges as a focal point in understanding TBI pathogenesis. Deciphering the complex interplay between microbial dysbiosis, ENS disruption, EEC dysfunction, ANS dysregulation and intestinal immune cell activation is crucial to develop successful TBI therapies, which have been elusive for decades. This entails understanding the temporal changes in immune system activation across the brain and the gut, as well as elucidating the effects of gut microbial metabolites and hormones, and the role of autonomic nervous system and systemic inflammation in the pathophysiological process of acute and chronic TBI.

## Data Availability

Not applicable.

## Abbreviations

TBI: Traumatic brain injury
GI: Gastrointestinal system
ENS: Enteric nervous system
ANS: Autonomic nervous system
ICP: Intracranial pressure
DAMP: Damage-associated molecular pattern
ROS: Reactive oxygen species
BBB: Blood–brain-barrier
AQP4: Aquaporin 4
ATP: Adenosine triphosphate
Bcl-2: B-cell lymphoma 2
ICU: Intensive care unit
HPA: Hypothalamic–pituitary–adrenal
Ccr2: C–C chemokine receptor type 2
TNF-α: Tumor necrosis factor alpha
IL-1β: Interleukin-1 beta
TLR4: Toll-like receptor 4
EGC: Enteric glial cell
Ach: Acetylcholine
α7nAChR: α7 Subtype of the nicotinic acetylcholine receptor
EEC: Enteroendocrine cell
VNS: Vagus/Vagal nerve stimulation
IBD: Inflammatory bowel disease
IBS: Irritable bowel syndrome
CNS: Central nervous system
GLP-1: Glucagon-like peptide 1
ChgA: Chromogranin A
Lgr5: Leucine-rich repeat-containing G-protein coupled receptor 5
Notch1: Notch receptor 1
Atoh1: Atonal bHLH transcription factor 1
Neurog3: Neurogenin 3
TPH: Tryptophan hydroxylase
SSRI: Selective serotonin reuptake inhibitor
SNRI: Serotonin-norepinephrine reuptake inhibitor
ICC: Interstitial cells of Cajal
IP: Intraperitoneal
FGF: Fibroblast growth factor
FGF-BP: FGF-binding protein
bFGF: Basic FGF
PI3K: Phosphoinositide 3-kinase
PIP3: Phosphatidylinositol 3,4,5 trisphosphate
PIP2: Phosphatidylinositol 3,4 bisphosphate
Pkb/Akt: Protein kinase B
IRI: Ischemia–reperfusion injury
SCI: Spinal cord injury
mTOR: Mammalian target of rapamycin
WD: Weight drop
ICH: Intracerebral hemorrhage
SMA: Superior mesenteric artery
BDNF: Brain-derived neurotrophic factor
CD55: Complement decay-accelerating factor
Nrf2: Nuclear factor erythroid 2-related factor 2
ICAM-1: Intercellular adhesion molecule-1
SAH: Subarachnoid hemorrhage
NE: Norepinephrine
HSP-70: Heat shock protein 70
UCHL-1: Ubiquitin carboxyl-terminal hydrolase L1
ZO-1: Zonula Occludens-1
vBNST: Ventral bed nucleus of the stria terminalis
AD: Alzheimer’s disease
CD68: Cluster of differentiation 68
GFAP: Glial fibrillary acidic protein
VEGF: Vascular endothelial growth factor
GSK-3beta: Glycogen synthase kinase-3beta
CD40: Cluster of differentiation 40
VCAM-1: Vascular cell adhesion molecule-1
SCFA: Short-chain fatty acid
Cb: *Clostridium* *butyricum*
LA: *Lactobacillus* *acidophilus*
